# The Importance of the Maxillary and Mandibular Incisors in Predicting the Canines and Premolars Crown Widths

**DOI:** 10.1155/2022/1551413

**Published:** 2022-06-18

**Authors:** Mohammed Rafid A. Al-Khannaq, Mohammed Nahidh, Dunia Ahmed Al-Dulaimy

**Affiliations:** ^1^Department of POP, College of Dentistry, Mustansiriyah University, Baghdad, Iraq; ^2^Department of Orthodontics, College of Dentistry, Universityof Baghdad, Baghdad, Iraq

## Abstract

**Background:**

The purpose of the present study was to use the combined mesiodistal crown widths of the mandibular and maxillary incisors as predictors for the combined mesiodistal crown widths of mandibular and maxillary canines and premolars.

**Materials and Methods:**

One hundred and twenty pairs of study models belong to 120 Iraqi adult subjects with normal dental and skeletal relations were included in the study. The crown widths of the mandibular and maxillary incisors, canines, and premolars were assessed at the maximum mesiodistal dimension on the dental casts using a digital electronic caliper with 0.01 mm sensitivity. The correlation between combined mesiodistal crown widths of the mandibular and the maxillary incisors and combined mesiodistal crown widths of mandibular and maxillary premolars and canines has been determined using Pearson's coefficient correlation test for each arch and gender. Using simple regression analysis, the equations predicting the widths of the mandibular and maxillary premolars and canines were established. The predicted and the actual mesiodistal crown width values have been compared with the use of a paired sample *t*-test.

**Results:**

According to the findings of the present study, males had significantly wider teeth compared to females. Correlations between the measured parameters ranged from moderate to strong. A nonsignificant difference between actual and predicted mesiodistal crown widths was discovered.

**Conclusions:**

With a high degree of accuracy, the combined mesiodistal widths of the maxillary and the mandibular incisors could be utilized for predicting the combined mesiodistal crown widths of the mandibular and maxillary canines and premolars.

## 1. Introduction

Diagnosing dental arch length insufficiency throughout the mixed dentition stage has been considered critical for preventing malocclusion. The mandibular and maxillary anterior teeth and first molars erupted during this stage along with the primary molars and canines. Following normal exfoliation of the primary molars and canines, crowding might arise [1].

Despite the fact that these teeth have larger widths compared to their successors along with the primary spaces, crowding might develop as a result of the large size of the teeth (i.e., determined genetically) or a lack of size in the dental arches as a result of an early loss of primary teeth or an increase in the trend toward soft foods, causing dental arches to not develop adequately enough to accommodate the entire set of the teeth (i.e., environmental aspect) [2].

Dental caries and consequent early extraction of primary teeth will lead to space loss of the erupting cuspids and bicuspids and the development of malocclusion [3], so maintaining oral hygiene measures will lessen this problem in children, allowing teeth to erupt in a normal situation. Preventive and educational programs in addition to the curative programs must be implemented to improve the dental care of children at the stage of primary and mixed dentition. These programs might include surveys, educational videos on YouTube a and developing oral health impact scale to diagnose such a problem and manage it early [4, 5].

Various approaches for predicting the width values of the permanent canines and the premolars have been devised to aid in the management of dental arch space. The teeth's average widths were initially published by Black [6]. As a result of individual differences in teeth sizes in various racial groupings and genders, Black's work has been less precise. Tanaka and Johnston [7] in 1974 and Moyers [8] in 1988 created the two most widely used approaches for predicting unerupted canines and premolars.

Tanaka and Johnston [7] had predicted mesiodistal width of unerupted premolars and canines in one quadrant through the addition of 11 mm (maxillary arch) as well as 10.5 mm (mandibular arch) to half of the total width of mandibular four incisors that have been evaluated from the dental cast.

Moyers [8], however, created a chart to predict the widths of the premolars and canines based upon the sum of the widths of the mandibular incisors. Those approaches might not be appropriate for all racial groups since the results could be overestimated.

Different approaches [9–25] used regression analyses to determine whether the width values of mandibular or maxillary anterior teeth and first molars were acceptable in various racial groupings.

Various studies were conducted in Iraq aiming at predicting the width values of premolars and canines with the use of many approaches [26–32]. This work aims to create new regression equations that could estimate the summation mesiodistal crown dimensions of premolars and canines by the use of combined incisor crown widths.

## 2. Materials and Methods

### 2.1. Sample

This retrospective study was authorized by the University of Baghdad's College of Dentistry's scientific and ethical committees. One hundred and twenty pairs of study models belong to 120 Iraqi Arabs (60 females and 60 males) aged between 17 and 25, with a full complement of the permanent teeth (excluding third molars), class I skeletal and dental relationships [2], and no history of bad oral habits, orthodontic treatments, defects, or maxillo-facial surgeries. Restorations, caries, malformations, and attrition are not present on the teeth.

### 2.2. Methods

**Figure 1:**
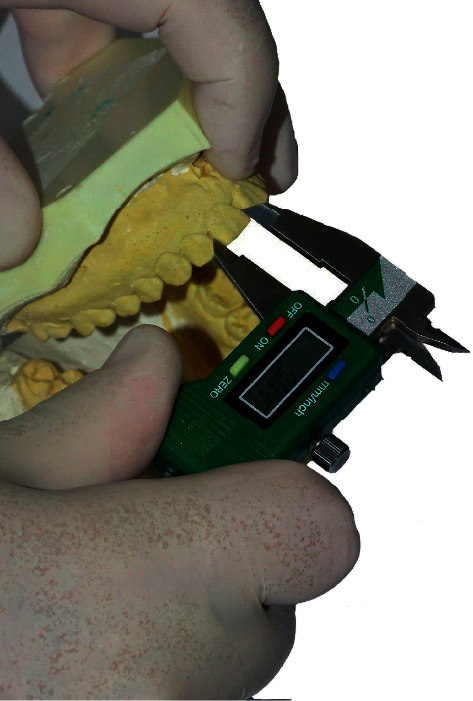
Measuring procedure.

Stone models of the chosen sample were acquired from the archives of the orthodontic department at the University of Baghdad's School of Dentistry. The mandibular and maxillary teeth (except for the first and second molars) have been measured at their maximum mesiodistal dimension using electronic digital calipers (Mitutoyo, Japan) with a 0.01 mm sensitivity (Figure 1).

### 2.3. Statistical Analysis

Using the SPSS program (version 25), the data were subjected to automated statistical analyses. Among the statistical analyses were:

(1) Descriptive statistics (means, standard deviation, and numbers and percentage values of cases that overestimated and underestimated actual width values of the canines and the premolars).
(2) Inferential statistics, which included
  - Shapiro–Wilk test was used to test the normality of data distribution.
  - To test intra- and interobserver reliability, the intraclass coefficient of correlation was used.
  - The correlation between combined mesiodistal widths of the mandibular and the maxillary premolars and canines and combined mesiodistal widths of the maxillary and mandibular incisors was determined using Pearson's coefficient test of correlation (*r*).
  - Independent sample *t*-test was used for testing the existence of the gender differences.
  - Simple regression analyses also used to find regression equations for the prediction of combined mesiodistal widths of the mandibular and maxillary premolars and canines.
  - In both genders, a paired sample *t*-test has been utilized to show if there was a significant difference between the actual and predicted mesiodistal widths of the maxillary and the mandibular premolars and canines.

A significance level of more than 0.05 indicates a nonsignificant difference or correlation.

## 3. Results

The Shapiro–Wilk test was used to determine the normality of data distribution, and the findings indicated that the data were normally distributed (*p* > 0.05).

The measurements were repeated after two weeks to test the intra- and interobserver reliability using an intraclass correlation test, and the results indicated high reliability (more than 0.9).

The descriptive statistics and gender differences for the combined mandibular and maxillary four incisors, premolars, and canines crown widths have been listed in Table 1. In general, the males had significantly higher mean values compared to females.

Table 2 shows the correlations between maxillary and mandibular incisors' combined widths, and mandibular and maxillary canines and premolars combined widths for both genders. Between them, there was a moderate to strong direct significant high correlation.

Table 3 shows the regression equations for the two genders that estimated the combined widths of the mandibular and maxillary premolars and canines from the total mesiodistal widths of the mandibular and maxillary incisors.

In Table 4, the actual combined widths of the maxillary and mandibular premolars and canines in both genders were compared with those predicted by regression equations. The predicted and actual measurements were found to differ by a nonsignificant amount.

Table 5 shows the percentages and numbers of cases in which the widths of permanent premolars and canines were underestimated, overestimated or lie within 2 mm difference in both genders and arches. With a high precision prediction of more than 83%, the data indicated a small number of cases that did not lie inside the 2 mm limit.

## 4. Discussion

Predictions of the combined widths of the mandibular and maxillary premolars and canines must be undertaken throughout the mixed dentition period to prevent the development of crowding in dental arches. Tanaka and Johnston [7] and Moyers [8] devised approaches to predict such teeth widths. Because of the racial differences in tooth sizes, such procedures cannot be used on all ethnic groups.

Various published articles attempted to predict the width of premolars and canines using a prediction approach based on the first permanent incisors and molars, which erupted early in life [9–25]. Caries can often impair the first molars, resulting in an eventual loss in children at nine to ten years old. At the same time, permanent incisors could be affected by different anomalies that may influence their number, shape, size, and position, yet they are less affected by caries [33–35].

Various studies [26–32] have used the prediction approach to develop the regression equations, which can predict the combined widths of the mandibular and maxillary premolars and canines in Iraqi samples. Since they have erupted early in the oral cavity, mandibular and maxillary incisors were selected for the first time to predict the widths of maxillary and mandibular premolars and canines in this research.

For verifying the gender difference, the first stage in statistics, as shown in Table 1, was to demonstrate the mean and standard deviation of the combined widths of the mandibular and maxillary incisors and the widths of the maxillary as well as mandibular premolars and canines. According to the findings, all of the assessed variables showed significant gender differences. This is consistent with the results of numerous previous researches [27, 28, 30–32]; therefore, the data were divided into females and males and analyzed independently.

The relationship between the combined mesiodistal crown dimensions of the mandibular and maxillary incisors and the combined mesiodistal crown dimensions of the mandibular and maxillary canines and premolars was tested in the second step. Table 2 showed that the measured variables had a moderate to strong direct high significant correlation, consistent with the prior findings [27, 28, 30–32].

The development of regression equations was the third phase. The equation is *Y* = *a* + *bX*, in which “*Y*” represents the combined mesiodistal crown widths of the maxillary and the mandibular permanent canines and premolars (each arch alone), “*X*” represents the combined mesiodistal crown of maxillary and mandibular incisors, “*a*” represented the constant, and “*b*” represented the coefficient of the regression. Table 3 shows equations for the two genders and arches.

After computing the predicted widths, the fourth step was to use the paired sample *t*-test for comparing the predicted and actual measurements. The findings revealed no statistically significant differences between actual and expected mesiodistal crown dimensions regarding both mandibular and maxillary premolars and canines (Table 4), which is consistent with previous research [21–25, 30–32]. The mean differences between the two approaches and their standard deviations are quite small and not clinically significant. Ideally, there should be no variation in the prediction approach between the actual and predicted widths of the permanent canines and premolars.

Prediction techniques are not always 100% accurate, and they could underestimate or overestimate the size of unerupted teeth. Overestimation appears to be the best way to avoid a lack of space; however, this method may mean tooth extractions for specific individuals. An overestimation of just 1 mm beyond actual width values regarding permanent canines and premolars on every one of the sides of the arch would have no significant impact on the choice to extract or not extract [8, 19, 21].

Table 5 shows the percentages and numbers of cases that overestimated and underestimated the actual width values of the permanent premolars and canines within the 2 mm limit (both mandibular and maxillary archsides, both genders). The data revealed a small number of cases that did not fall inside the 2 mm precision limit on the two sides, with a precision rate of more than 83%. This, along with the good selection of the teeth that have been utilized in the predictions, the excellent correlation between the variables, and the nonsignificant approach difference, is regarded as a strong point for this prediction approach.

The sample size was a crucial drawback in this research because getting a sample with normal occlusion and sound teeth is so difficult. With the development in technology and presence of special analyzing software, intraoral scanner devices, digital camera, and CBCT, it became easier to measure teeth and dental arch dimensions in three planes of space [36]. This replaces the use of conventional methods of measuring these parameters. Moreover, diagnosis, exchange of clinical information, simulating treatment plans and obtaining dental consultation have become far more feasible by these means, especially during the COVID-19 pandemic [37]. Hence developing a special software for prediction using neural networks is required for diagnosis and implementing treatment planning.

## 5. Conclusions

The differences between the actual and predicted mesiodistal crown width values were nonsignificant. As a result, because no radiographs are needed, and it is based upon eight permanent teeth which emerge early in life, the summation of the mesiodistal widths of maxillary and mandibular incisors could be utilized for predicting the combined mesiodistal widths of the mandibular and maxillary canines and premolars with high reliability.

## Figures and Tables

**Table 1:** Descriptive statistics and gender difference for the variables measured.

Variables	Genders	Descriptive statistics			Gender difference	
		*N*	Mean	S.D.	*t* test	*p* value
Sum of upper and lower incisors crown width	Males	60	54.065	2.884	4.372	*0.001*
	Females	60	51.949	2.396		

Sum of upper canines and premolars crown width	Males	60	43.464	1.884	4.491	*0.001*
	Females	60	41.880	1.979		

Sum of lower canines and premolars crown width	Males	60	42.492	1.844	4.494	*0.001*
	Females	60	40.973	1.858		

p value ≤ 0.01 is considered highly significant.

**Table 2:** Correlation among the measured variables.

Genders	Correlation	Upper	Lower
Males	*r*	0.682	0.727
	*p*	*0.001*	*0.001*

Females	*r*	0.622	0.673
	*p*	*0.001*	*0.001*

p value ≤ 0.01 is considered highly significant.

**Table 3:** Regression equations used to predict the sum of upper and lower canines and premolars crown widths in both genders.

Genders	Upper	Lower
Males	*Y* = 19.362 + 0.446*X*	*Y* = 17.350 + 0.465*X*

Females	*Y* = 15.179 + 0.514*X*	*Y* = 13.856 + 0.522*X*

**Table 4:** Descriptive statistics and methods difference in both genders.

Genders	Variables	Descriptive statistics				Method difference		
		Actual measurements		Predicted measurements				
		Mean	S.D.	Mean	S.D.	Mean difference	*t* test	*p* value
Males	Sum of upper canines and premolars crown width	43.464	1.884	43.475	1.286	−0.011	−0.061	0.952
	Sum of lower canines and premolars crown width	42.492	1.844	42.490	1.341	0.002	0.011	0.991

Females	Sum of upper canines and premolars crown width	41.880	1.979	41.881	1.232	−0.001	−0.005	0.996
	Sum of lower canines and premolars crown width	40.97333	1.858	40.97329	1.251	0.00004	0.0002	0.999

**Table 5:** The numbers and percentages of cases that lie within the limit of 2 mm, over an underestimated actual combined width of canines and premolars in both arches and genders.

Genders	Males			Females		
Arches	Within the limit	Overestimation	Underestimation	Within the limit	Overestimation	Underestimation
Maxillary	52 (86.8%)	4 (6.6%)	4 (6.6%)	50 (83.3%)	4 (6.7%)	6 (10%)
Mandibular	54 (90%)	2 (3.3%)	4 (6.67%)	50 (83.3%)	4 (6.7%)	6 (10%)

## Data Availability

The data used to support the findings of this study are available from the corresponding author upon request.
